# Salidroside alleviates LPS-induced liver injury and inflammation through SIRT1- NF-κB pathway and NLRP3 inflammasome

**DOI:** 10.22038/IJBMS.2023.69401.15124

**Published:** 2024

**Authors:** Jialei Meng, Yunfeng Li, Fangyuan Sun, Wentao Feng, Hui Ye, Tianning Tian, Ming Lei

**Affiliations:** 1 Trauma Emergency Center, Seventh People’s Hospital of Shanghai University of Traditional Chinese Medicine, No.358, Datong Road, Pudong New District, Shanghai 200137, China; # These authors contributed eqully to this work

**Keywords:** Anti-inflammation, Lipopolysaccharide, Liver injury, Nuclear factor Kappa B, Salidroside

## Abstract

**Objective(s):**

Salidroside (SAL), an active ingredient purified from the medicinal plant Rhodiola rosea, has anti-inflammatory, anti-oxidant, anticancer, and neuroprotective properties. The study aims to examine SAL’s protective role in liver damage brought on by lipopolysaccharide (LPS).

**Materials and Methods:**

Six to eight-week-old male C57BL/6 wild-type mice were intraperitoneally treated with 10 mg/kg LPS for 24 hr and 50 mg/kg SAL two hours before LPS administration. Mice were categorized into control, LPS, and LPS + SAL groups. To evaluate liver injury, biochemical and TUNNEL staining test studies were performed. The Elisa assay analyzed interleukin- 1β (IL-1β), tumor necrosis factor-alpha (TNF-α), and interleukin-6 (IL-6) pro-inflammatory cytokine expression levels. RT-qPCR and western blotting measured mRNA and protein expression of SIRT1, NF-кB, NLRP3, cleaved caspase-1, and GSDMD, respectively.

**Results:**

Analysis of the serum alanine/aspartate aminotransferases (ALT/AST), malondialdehyde (MDA), superoxide dismutase (SOD), and catalase (CAT) revealed that SAL protected against hepatotoxicity induced by LPS. The pathological evaluation of the liver supported the protection provided by SAL. SAL treatment reversed IL-1β, TNF-α, and IL-6 pro-inflammatory cytokines after being induced by LPS (all, *P*<0.001). The western blotting examination results demonstrated that SAL increased the levels of Sirtuin 1 (SIRT1) expression but markedly reduced the phosphorylation of Nuclear Factor Kappa B (NF-B) and the expressions of NLRP3, cleaved caspase-1, and gasdermin D (GSDMD) induced by LPS (all, *P*<0.001).

**Conclusion:**

Our results speculated that by inhibiting the SIRT1- NF-κB pathway and NLRP3 inflammasome, SAL defends against LPS-induced liver injury and inflammation.

## Introduction

The most common reason for organ failure, including liver injury, is sepsis, a disorder characterized by systemic inflammatory dysfunction (1, 2). Sepsis has recently been described as the most common cause of mortality in intensive care units (ICUs) (3). The liver is particularly vulnerable to endotoxin-induced injury during sepsis because it involves several physiological and pathological processes, including metabolism, detoxification, homeostasis, and immunology (4). The common reason for mortality in sepsis patients is acute liver injury (ALI) brought on by the condition. Patients with ALI experience a wide range of symptoms, from mildly elevated liver enzymes to severe liver damage. The course of illness and mortality are closely linked to sepsis-induced liver damage (5). Apoptosis, oxidative stress, inflammatory and immune responses, and cellular hypoxia are some of the pathways that have been proposed to be engaged in the genesis of sepsis-associated ALI (6). However, sepsis-induced liver impairments underlying cause and course remain unknown.

Several investigations have shown that Gram-negative bacteria produce lipopolysaccharide (LPS), which has been linked to sepsis. It has the potential to cause the development of organ failure, including liver failure (2). The connection between LPS-induced endotoxemia and liver injury has been shown in several experimental animal models (7). The liver is the most significant immunological and detoxifying organ in the human body, and liver dysfunction causes LPS to operate as an active hepatic endotoxin. Furthermore, LPS increases the production of inflammatory cytokines by hepatic Kupffer cells (macrophages) and triggers hepatocyte necrosis or apoptosis, resulting in liver injury (8). However, no studies have been conducted to examine how inflammation and apoptosis may contribute to liver damage brought on by sepsis.

Salidroside (SAL) is an active constituent derived from *Rhodiola rosea*, a medicinal herb that has traditionally been used to treat high-altitude sickness and weariness (9). Due to its many pharmacological attributes, such as anti-oxidant, anti-inflammatory, anticancer, and neuroprotection, SAL has lately drawn more attention (10-12). In mice induced by LPS or paraquat, SAL has been found to lessen acute lung damage (13, 14). Furthermore, sepsis and acute lung injury brought on by LPS are both prevented in mice by SAL (15, 16). In addition, to lessen pathological damage in concanavalina-induced liver injury, SAL can reduce liver enzyme levels and control inflammatory responses in the blood serum (17). SAL has potent free radical scavenging capabilities, contributing to its protective benefits against furan-induced hepatocyte injury (18). A previous study reported that in type 2 diabetic C57BLKS/Leprdb (db/db) mice, SAL could reduce hepatic steatosis (19). It is unknown, nevertheless, whether SAL protects against inflammation and damage to the liver brought on by LPS. In the current investigation, we planned a study on how SAL protects against inflammatory injury and associated pathways in LPS-induced liver damage.

## Materials and Methods

***Animals and treatment***

Six to eight-weeks old male C57BL/6 wild-type mice were kept in the SPF setting (ambient temperature, 22 ± 2 °C; humidity 40%), with a 12-hour light/dark cycle and access to food and drink. Mice were given 10 mg/kg LPS (L2630, Sigma) or an equivalent volume of saline intraperitoneally for 12 hr. Mice in Salidroside (SAL, HY-N0109, MedChemExpress) intervention group were also injected with SAL (50 mg/kg) 2 hr before LPS injection. Mice were categorized into the control, LPS, and LPS + SAL (50 mg/kg) groups, with eight mice in each group. Tail vein blood was collected at 6 hr. After 12 hr of LPS injection, mice were given a 2% isoflurane inhalation anesthesia (RWD, Shenzhen, China), after which blood and liver tissue samples were collected. The Ethics Committees of our hospital approved the experimental animal procedures.

***Biochemical analysis***

To evaluate the ALT (C009-2-1, Nanjing Jiancheng, China) and AST (C010-2-1, Nanjing Jiancheng, China) activities by colorimetry, serum was obtained and tested. In regular cold saline, the liver tissue was collected and homogenized (10%, w/v). Using commercially available kits, the lysate was collected to measure the MDA (S0131S, Beyotime, China) content, SOD (S0109, Beyotime, China), and CAT (S0051, Beyotime, China) activity.

***ELISA assay***

TNF-α (catalog number MTA00B, R&D Systems), IL-1β (catalog number MLB00C, R&D Systems), and IL-6 (catalog number M6000B, R&D Systems) concentrations in blood samples were assessed using ELISA kits. The cytokine concentrations were calculated using the pertinent standard curve after the 450 nm absorbance was obtained in a microplate reader.

***TUNEL staining***

The TUNEL apoptosis test kit was used to detect apoptosis in isolated cells. In 6-well plates, cells were grown and treated with 4% paraformaldehyde. Cells were first washed using PBS, then permeabilized with 0.3 percent Triton X-100, then treated for an hour at 37 °C with 50 L of TUNEL reaction fluid. DAPI was then used to stain the cell nuclei, and an inverted microscope (IX51, Olympus, Japan) was used to see the results.

***Detection of reactive oxygen species (ROS) production***

Dihydroethidium (DHE; Beyotime Institute of Biotechnology; cat. no. S0063) staining was used to identify cellular ROS formation. When superoxide anions enter living cells, they dehydrogenate DHE to produce ethidium, which then attaches to RNA or DNA to make red fluorescence. The higher the amounts of intracellular superoxide generation, the brighter the red fluorescence. Isolated cells were first seeded onto 24-well plates at a density of 1x10^5^ cells/well in this investigation. DHE (10 M) was then added to the cells and incubated for 30 min at 37 °C before being treated with 100 nM Se and 200 mM HCY for 24 hr at 37 °C. The fluorescence of the cells was studied under a fluorescent microscope at x100 magnification after they were rinsed with PBS.

***Reverse transcription quantitative (RT-q) PCR***

Mice atrial tissues were treated with TRizol (Invitrogen, USA) to extract the total RNA. Total RNA was converted into complementary DNA (cDNA) via reverse transcription. SYBR Green reagent (TaKaRa, Japan) was used in an ABI Prism 7700 Real-Time PCR equipment (Applied Biosystems, USA) to amplify the mRNA by Real-Time Quantitative PCR (RT-qPCR). The primer sequences that were employed are listed as follows: TNF-α (forward: 5′-GTG CCA GCC GAT GGG TTG TAC C-3′; reverse: 5′-AGG CCC ACA GTC CAG GTC ACT G-3′); IL-1β (forward: 5′-TTT GAA GTT GAC GGA CCC C-3′; reverse: 5′-GAT GTG CTG CTG CGA GAT T-3′); IL-6 (forward: 5′-AAC CAC GGC CTT CCC TAC T-3′; reverse: 5′-CAT TTC CAC GAT TTC CCA GA-3′); GAPDH (forward: 5′-TCA ACA GCA ACT CCC ACT CTT CCA-3′; reverse: 5′-ACC CTG TTG CTG TAG CCG TAT TCA-3′). The 2-ΔΔCq technique was utilized to examine the qPCR results (20).

***Western blotting***

Following protein extraction using RIPA buffer and centrifugation of the lysates to get supernatants, protein concentrations were determined using a BCA protein assay kit. Protein samples (50 μg) were electrophoresed on 10% SDS-PAGE before being transferred to a PVDF membrane (Millipore, Bedford, MA, USA). 5% skimmed milk dissolved in a TBS solution was then used to block the membrane. The membrane was incubated for the whole night at 4 °C against SIRT1 (1:400, sc-74465, mouse monoclonal, Santa Cruz), p-NF-кB (1:500, sc-166748, mouse monoclonal, Santa Cruz), NF-кB (1:500, sc-8008, mouse monoclonal, Santa Cruz), NLRP3 (1:400, ab214185, rabbit polyclonal, Abcam), Cleaved caspase-1 (1:400, #89332, rabbit monoclonal, Cell Signaling Technology), GAPDH (1:1000, ab9485, rabbit polyclonal, Abcam), and GSDMD (1:500, ab209845, rabbit monoclonal, Abcam). Secondary antibodies conjugated to HRP (1:2000) were used to identify the immunological reactivity of these target proteins. The protein band was observed using ECL (Thermo, Waltham, MA, USA), and band density was measured using ImageJ software (Bio-Rad, Hercules, CA, USA).

***Statistical analysis***

The study was done using SPSS 20.0 (CA, USA) statistical program, and the data of the results were reported as means ± standard deviation (SD). Data differences among the three groups were evaluated with a one-way analysis of variance (ANOVA), followed by Tukey’s test.  *P*<0.05 was used in this investigation to determine if the differences were significant.

## Results

***SAL reduced the severity of the sepsis-induced liver injury***

**Figure 1:**
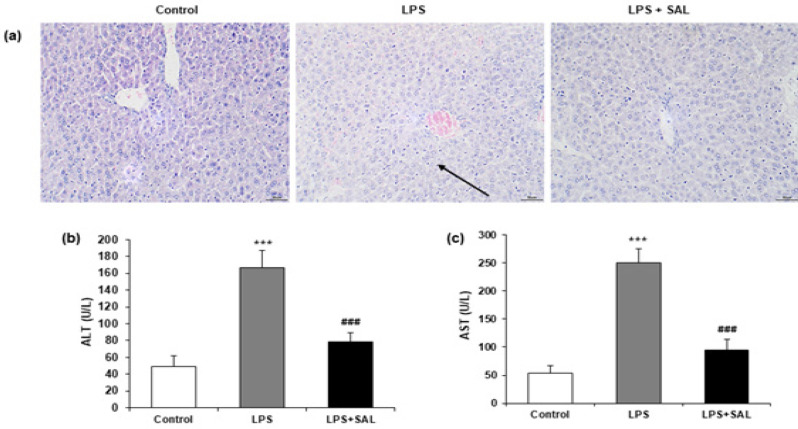
SAL reduces LPS-induced liver damage

To investigate the impact of SAL on sepsis-induced liver damage, HE staining was carried out in the liver tissue of mice. Liver cells were arranged erratically after receiving LPS therapy, and the hepatic lobule structure was aberrant. SAL administration ameliorated inflammatory cell infiltration, necrosis, and degeneration (Figure 1a). Six hours following LPS injection, two serum aspartate aminotransferase (AST) and alanine aminotransferase (ALT) concentrations were also assessed as indicators of liver function. The levels of serum AST and ALT were potentially elevated in the LPS group compared with the control group (all, *P*<0.001). SAL mitigated the LPS-induced increase in serum ALT and AST levels (all, *P*<0.001) (Figure 1b, c).

***SAL inhibited the production and transcription of LPS-induced proinflammatory factors***

**Figure 2:**
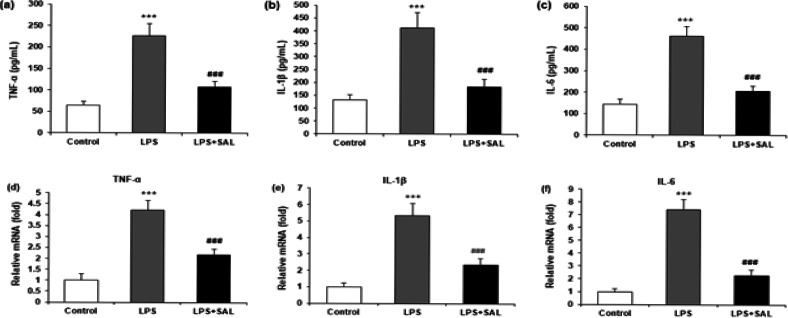
Impact of SAL on inflammatory factors in serum and cell supernatants. (a, b, c) ELISA was performed to measure each group's serum levels of TNF-α, IL-1β, and IL-6. (d, e, f) RT-qPCR was performed to determine the mRNA levels of TNF-α, IL-1β, and IL-6 genes in liver tissues of each group

Changes in proinflammatory cytokines were evaluated using an ELISA assay and RT-qPCR to investigate SAL’s anti-inflammatory effect. Serum levels of TNF-α, IL-1β, and IL-6 were markedly enhanced in the LPS group (all, *P*<0.001), and SAL cotreatment significantly inhibited these changes (all, *P*<0.001) (Figure 2a-c). Meanwhile, mRNA levels of TNF-α, IL-1β, and IL-6 in liver tissues were elevated after LPS treatment (all, *P*<0.001), and the effects were also mitigated by SAL intervention (all, *P*<0.001) (Figure 2d-f).

***SAL inhibited LPS-induced apoptosis in liver tissues***

**Figure 3:**
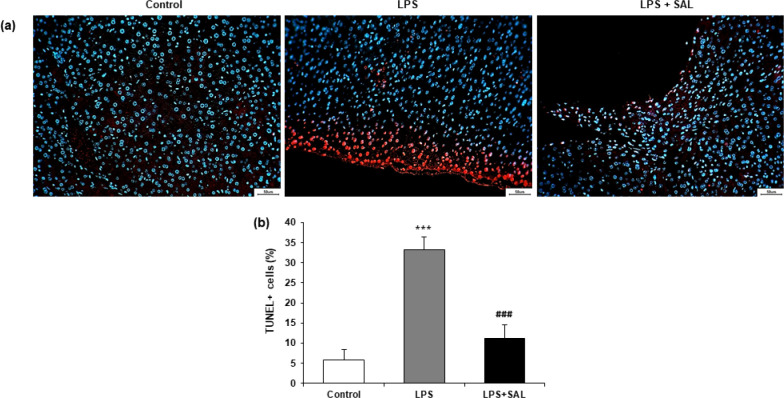
SAL reduces liver tissue apoptosis that is induced by LPS

To determine if apoptosis is involved in the inhibitory impact of SAL on liver damage, TUNEL staining was used on the liver tissue of mice. LPS stimulation significantly promoted apoptosis, as evidenced by obviously enhanced green fluorescence (TUNEL+) in the mice’s liver tissue. These pro-apoptotic effects of LPS were markedly reversed after SAL treatment (Figure 3a). SAL considerably decreased the proportion of TUNEL+ cells in the LPS-induced mice’s liver tissue, according to quantification analysis (*P*<0.001) (Figure 3b).

***SAL reduced ROS injury in sepsis-induced liver injury***

**Figure 4:**
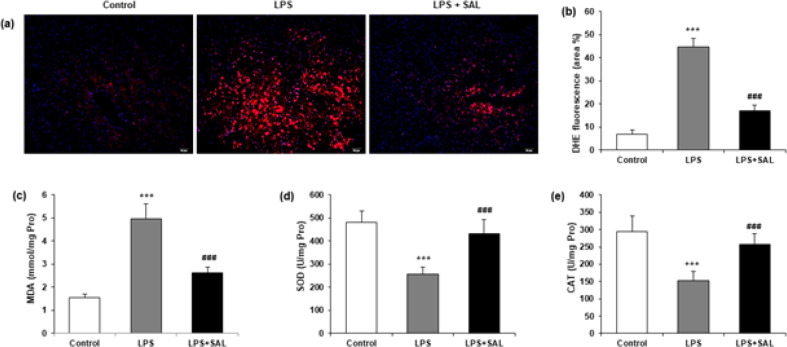
SAL reduces LPS-stimulated ROS generation and oxidative stress

The dihydroethidium (DHE) staining showed that LPS administration significantly enhanced fluorescence intensity compared to the control group. The rise in DHE fluorescence intensity caused by LPS was significantly reduced by SAL (Figure 4a, b). Moreover, SAL also showed an inhibitory effect on LPS-induced oxidative stress. The malondialdehyde (MDA) level was lower, and the superoxide dismutase (SOD) and catalase (CAT) activities were higher in the LPS + SAL group compared to the LPS group (Figure 4c-e) (all, *P*<0.001).

***SAL modulated the SIRT1/NF-кB pathway in sepsis-induced liver injury***

**Figure 5:**
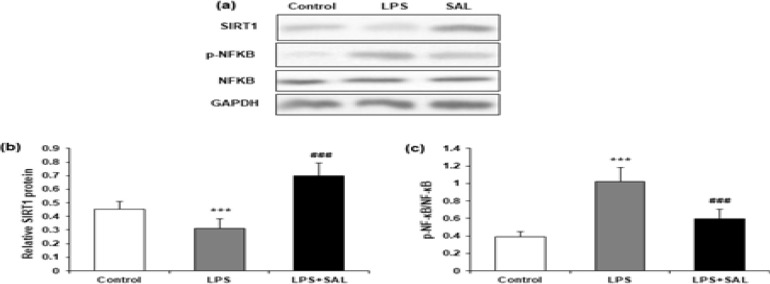
Effects of SAL on SIRT1 and NF-кB

The protein expression of SIRT1 and NF-кB in mice liver tissue was examined using western blot (Figure 5a). SIRT1 expression in liver tissue was reduced by LPS and then elevated by SAL cotreatment (*P*<0.001) (Figure 5b). In addition, SAL significantly mitigated the LPS-induced phosphorylation of NF-кB (normalized to total NF-кB protein) (*P*<0.001) (Figure 5c).

***SAL suppressed LPS-promoted NLRP3 inflammasome***

**Figure 6:**
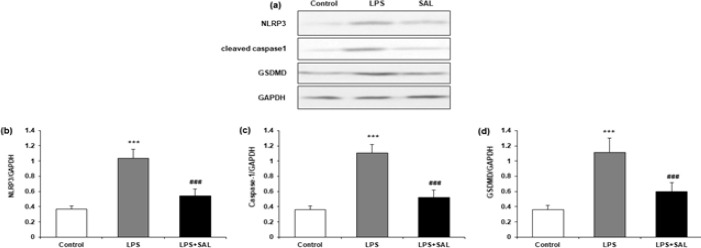
Effects of SAL on the NLRP3 pathway caused by LPS

To determine the NLRP3 protein levels and associated proteins, we employed western blotting (Figure 6a). NLRP3, cleaved caspase-1, and GSDMD protein expression were all higher in LPS-treated cells compared to control cells, providing conclusive evidence that LPS-activated the NLRP3 inflammasome. The enhanced expression of these proteins caused by LPS was reversed by SAL cotreatment (Figure 6b-d).

Mice were pretreated with SAL (50 mg/kg) for 2 hr and then intraperitoneally injected with LPS (10 mg/kg). a Images of the liver tissues in each group stained with HE are representative. The black arrow indicates the infiltrated inflammatory cells; b, c serum ALT and AST levels were measured at 6 hr after intraperitoneal injection of LPS. The results are shown as mean ± SD (8 mice per group). ***P<0.001 vs control group; ###*P*<0.001 vs LPS group.

SAL: Salidroside; LPS: lipopolysaccharide; ALT and AST: Alanine/aspartate aminotransferases

The results are shown as mean ± SD (8 mice per group). ***P<0.001 vs control group; ###*P*<0.001 vs LPS group.

SAL: Salidroside

a Representative images of TUNEL staining for each group. b Number of TUNEL-positive cells. Results are shown as mean ± SD (8 mice per group). ***P<0.001 vs control group; ###*P*<0.001 vs LPS group

SAL: Salidroside; LPS: lipopolysaccharide

a DHE staining was applied to liver tissue to show ROS (red fluorescence). b Quantification of DHE fluorescence intensity. Levels of c MDA content, d SOD activity, and e CAT activity were assessed in liver tissue lysates (normalized to protein). The results are shown as mean ± SD (8 mice per group). ***P<0.001 vs control group; ###*P*<0.001 vs LPS group.

SAL: Salidroside; LPS: lipopolysaccharide; ROS: Reactive oxygen species; DHE: Dihydroethidium; MDA: Malondialdehyde; SOD: Superoxide dismutase; CAT: Catalase

a Representative gel blots showing SIRT1 levels, phosphorylated NF-кB, and total NF-кB (GAPDH as loading control). These protein blots were quantified for b SIRT1 (normalized to GAPDH) and c phosphorylated NF-кB (normalized to total NF-кB). The results are shown as mean ± SD (8 mice per group). ***P<0.001 vs control group; ###*P*<0.001 vs LPS group.

SAL: Salidroside; SIRT1: Sirtuin 1;LPS: lipopolysaccharide

a Representative NLRP3-related protein gel blots. Protein blots were quantified for b NLRP3, c Cleaved caspase-1, and d GSDMD. GAPDH was used as a control. Results are shown as mean ± SD (8 mice per group). ***P<0.001 vs control group; ###*P*<0.001 vs LPS group.

SAL: Salidroside; LPS:Lipopolysaccharide; GSDMD: Gasdermin D

**Figure 7:**
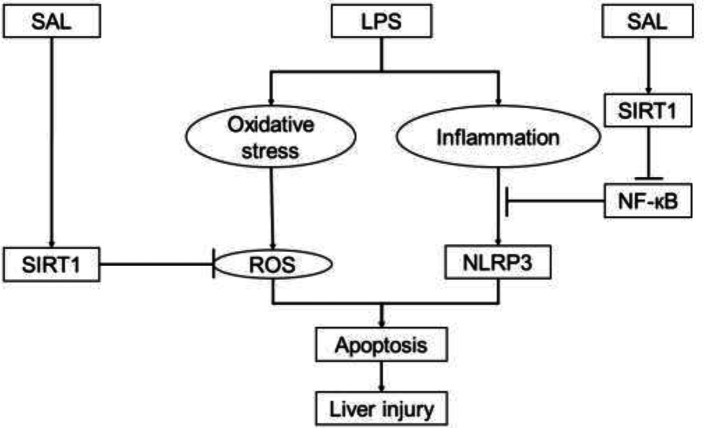
Model illustration on SAL-mediated regulation of SIRT1/NF-кB and NLRP3 pathways, which underlies the anti-inflammatory, anti-oxidant, anti-apoptotic, and anti-liver damage actions of LPS

SAL: Salidroside; LPS: lipopolysaccharide

## Discussion

In this current research, we evaluated the possible impact of SAL on LPS-induced liver damage and inflammation using the SIRT1-NF-κB pathway and the NLRP3 inflammasome. Our data showed that SAL potentially reduced the severity of sepsis-induced liver injury. SAL inhibited the production and transcription of LPS-induced proinflammatory factors, apoptosis in liver tissues, and decreased ROS injury in sepsis-induced liver damage. Further, SAL significantly increased SIRT1 expression (*P*<0.001), whereas it significantly attenuated LPS-induced phosphorylation of Nuclear Factor Kappa B (NF-κB) as well as expression of NLRP3, cleaved caspase-1, and gasdermin D (GSDMD) (all, *P*<0.001). Thus, the results speculate that SAL could have a protective effect on LPS-induced liver injury and inflammation.

SAL is an active ingredient produced from *Rhodiola rosea*, a medicinal plant widely used to alleviate fatigue and high-altitude sickness (9). Recent research evidence demonstrates that SAL is effective in treating anti-inflammatory, anti-oxidant, anticancer, and in neuroprotection (10-12). In the present investigation, we found that SAL pre-treatment (50 mg/kg) significantly reduced LPS-induced liver damage, as demonstrated by decreased ALT and AST levels (all, *P*<0.001) (Figure 1b, c), as well as reducing histological abnormalities (Figure 1a). Therefore, SAL reduced the degree of liver damage brought on by sepsis.

Cytokines are crucial in host defense and the pathophysiological development of infectious and inflammatory diseases. LPS is a potent activator of innate immune cells and induces the production of pro-inflammatory cytokines (21). It is well established that TNF-α, IL-1β, and IL-6 are significant inflammatory mediators. Our study investigated the expression of TNF-α, IL-1β, and IL-6 pro-inflammatory cytokines using LPS-induced liver tissues. The results demonstrated that serum TNF-α, IL-1β, and IL-6 levels were potentially enhanced in the LPS group (all, *P*<0.001). In contrast, SAL cotreatment potentially attenuated TNF-α, IL-1β, and IL-6 expression (all, *P*<0.001) (Figure 2a-c). Previous research reported that TNF-α, IL-1β, and IL-6 expression levels were significantly enhanced after LPS treatment, and SAL could attenuate TNF-α, IL-1β, and IL-6 production in LPS-treated mice serum (22), which is consistent with our present study. Therefore, in the LPS-treated mice model, our results confirmed the protective effect of SAL in anti-inflammation.

Cell apoptosis plays a crucial role in tissue homeostasis and embryonic development, and it can trigger a series of pathogenic events under certain circumstances (23). Hepatic stellate cell (HSC) activation, liver fibrosis, and inflammation were all exacerbated by the injured hepatocytes’ tendency to apoptosis (24). Mainly extrinsic and intrinsic mechanisms are involved in cell apoptosis. The Bcl-2 family controls the intrinsic or mitochondrial-mediated apoptosis pathway (25). However, our present study observed LPS-induced apoptosis in liver tissues. Increased green fluorescence (TUNEL+) in the mouse liver tissue demonstrated that LPS stimulation extensively promoted apoptosis. These pro-apoptotic effects of LPS were significantly reversed after SAL treatment (Figure 3a). SAL considerably reduced the proportion of TUNEL+ cells in the liver tissue of LPS-treated mice, according to quantification analysis (*P*<0.001) (Figure 3b).

According to an increasing number of studies, herbal drugs derived from plant extracts are increasingly being used to treat liver disease (26). Through a variety of intracellular signaling channels, plant extracts aid in reducing inflammatory responses in hepatocytes. Several natural compounds, such as andrographolide, chlorogenic acid, and quercetin, have been reported to have anti-oxidant properties that help to reduce drug-induced liver injury (DILI) (27-29). Our findings showed that SAL raised SOD and CAT levels and decreased MDA levels induced by LPS (all, *P*<0.001) (Figure 4c-e), demonstrating that SAL may attenuate oxidative liver damage and hence ameliorate LPS-induced liver injury. 

Two crucial signaling molecules in proinflammatory signaling are reactive oxygen species (ROS) and nuclear factor-kB (NF-kB). Endogenous and external stimuli can both produce the former. Through its activation, the latter can effectively control the production of proinflammatory cytokines. Its excessive accumulation can lead to oxidative stress damage and an imbalance of intracellular redox processes. Furthermore, there is a strong link between ROS and NF-kB signaling activity (30, 31). According to recent research, ROS is a crucial trigger for the development and activation of inflammasomes (32). Our results demonstrated that SAL could attenuate oxidative stress and the development and activation of the NLRP3 inflammasome induced by LPS, which is consistent with the previous study. The fluorescence results showed that SAL could diminish the ROS signal caused by LPS. LPS-induced phosphorylation of NF-kB was also dramatically reversed (Figure 5c). Therefore, we hypothesized that SAL might inhibit inflammasome activation by lowering ROS generation and NF-kB phosphorylation and perform an anti-inflammatory function in LPS-induced sepsis (Figure 7).

## Conclusion

This work examined the protective effects of SAL against LPS-induced liver inflammation and damage. Our findings showed that SAL’s hepatoprotective properties are due to its significant anti-inflammatory and anti-oxidative effects caused by activating the SIRT1/NF-кB pathway and NLRP3 Inflammasome. Accumulating evidence speculated that SAL could be a potential therapeutic agent for sepsis-induced liver injury. 

## Authors’ Contributions

M L designed and supervised the project and revised the manuscript. J M and Y L performed experiments and wrote the first draft of the manuscript. F S, W F, and H Y helped perform the experiments and collect data. T T analyzed the data and performed the statistical analysis.

## Statement of Ethics

All experimental protocols were approved by the Experimental Animal Ethics Committee of the Seventh People’s Hospital of Shanghai University of Traditional Chinese Medicine. All methods were carried out in accordance with the National Institute of Health Guidelines for Care and Use of Laboratory Animals in Biomedical Research and reported in accordance with ARRIVE guidelines for the reporting of animal experiments.

## Funding Sources

This study was supported by 1) The National Natural Science Foundation of China (No. 81973649, 82174189). 2) Open Fund of the Key Laboratory of Emergency and Trauma Research of the Ministry of Education (Hainan Medical University) (Gant.KLET-202115). 3) Construction of Shanghai Municipal Health Commission East China Area and Municipal Traditional Chinese Medicine Specialized Disease Alliance (ZY (2021-2023) -0302). 4) Key Sub College of Shanghai Pudong New Area Health Commission (PWZy2020-07). 5) Shanghai Pudong New Area Health Commission Joint Public Relations Project (PW2021D-05). 6) Medical Discipline Construction in Pudong New Area, Shanghai (PWYgy2021-06). 7) Pudong New Area National Comprehensive Reform Pilot Zone for Traditional Chinese Medicine Development Project (PDZY-2022-0701).

## Data Availability Statement

All data generated or analyzed during this study are included in this article. Further inquiries can be directed to the corresponding author.

## Conflicts of Interest

The authors declared no conflicts of interest with other people or organizations.
